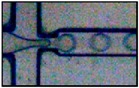# Fabrication of Glass Microchannel via Glass Imprinting using a Vitreous Carbon Stamp for Flow Focusing Droplet Generator

**DOI:** 10.3390/s18010083

**Published:** 2017-12-29

**Authors:** Hyungjun Jang, Muhammad Refatul Haq, Youngkyu Kim, Jun Kim, Pyoung-hwa Oh, Jonghyun Ju, Seok-Min Kim, Jiseok Lim

**Affiliations:** 1 School of Mechanical Engineering, College of Engineering, Chung-Ang University, 84 Heukseok-ro, Dongjak-gu, Seoul 06974, Korea; janghj@cau.ac.kr (H.J.); refat@cau.ac.kr (M.R.H.); kykdes@cau.ac.kr (Y.K.); zuhn@cau.ac.kr (J.K.); nanopeace@cau.ac.kr (P.-h.O.); jhju@cau.ac.kr (J.J.); 2 School of Mechanical Engineering, Yeungnam University, 280 Daehak-Ro, Gyeongsan, Gyeongbuk 38541, Korea

**Keywords:** autofluorescence, glass imprinting process, vitreous carbon mold, glass microchannel, droplet based microfluidic

## Abstract

This study reports a cost-effective method of replicating glass microfluidic chips using a vitreous carbon (VC) stamp. A glass replica with the required microfluidic microstructures was synthesized without etching. The replication method uses a VC stamp fabricated by combining thermal replication using a furan-based, thermally-curable polymer with carbonization. To test the feasibility of this method, a flow focusing droplet generator with flow-focusing and channel widths of 50 µm and 100 µm, respectively, was successfully fabricated in a soda-lime glass substrate. Deviation between the geometries of the initial shape and the vitreous carbon mold occurred because of shrinkage during the carbonization process, however this effect could be predicted and compensated for. Finally, the monodispersity of the droplets generated by the fabricated microfluidic device was evaluated.

## 1. Introduction

Microfluidic technology is a rapidly developing field of research that focuses on the manipulation of small quantities of fluids. It is a powerful tool for applications that require quantitative, high-throughput analysis in biochemistry, material science, and fluid physics [1,2,3,4,5]. The fabrication technologies used to make microfluidic devices are key to practical applications in this field.

One type of polymer microfluidic device is a poly-dimethylsiloxane (PDMS) part fabricated via soft lithography [6,7]. This device enables a relatively simple procedure for the replication and sealing of chips. In addition to the advantages of a convenient fabrication process, PDMS has high gas permeability and optical transparency, which are beneficial in a variety of biological applications. However, such elastomer-based polymer microfluidic devices have limitations [8,9,10,11] in many applications that require mechanical, thermal, and chemical stability. Another drawback is the limited applicability of PDMS in high-sensitivity fluorescence-based read-out applications, due to its high autofluorescence level [12]. Glass microfluidic devices may be an alternative for applications that require physical or chemical characteristics which are not offered by PDMS devices. Glass microfluidic devices provide exceptional chemical resistance, biocompatibility and optical properties, as well as mechanical stability, which prevents swelling and deformation [13,14,15,16]. In the realization of glass microfluidic chips, the pattering of the microfluidic channel is the most important issue. Selective material removal processes based on conventional semiconductor manufacturing techniques including photolithography and wet- or dry-etching are typically used to fabricate the glass microfluidic channel. Even with the advantages of glass, their high cost of fabrication limits their use, especially for disposable devices [17,18,19]. Therefore, considerable attention is focused in developing cost-effective fabrication solutions for glass microfluidic channels.

A glass imprinting process for micro/nano replication was recently studied using commercially available glasses such as quartz, Pyrex, and soda-lime glass [20,21]. This may be a promising approach to low-cost glass microchannel fabrication. The key issue in the mass production of glass microfluidic channels is the cost of the mold fabrication method, which must support small structures dispersed over a large area. This is because multiple molds are required in progressive glass imprinting systems which can provide a high production rate [22]. Since the mold used in the glass imprinting process must have superior thermal resistance, sufficient mechanical strength and good release properties, the selection of mold materials is limited. Some materials such as tungsten carbide, silicon carbide, nickel alloy, and glassy carbon [23,24,25,26] have been proposed for use in molds. Molds must be microfabricated at high resolution. However, the micro-texturing methods available for these materials are not suitable for large area applications because of their high processing costs and low throughput [27].

In this study, we propose a vitreous carbon (VC) mold, made via a combination of replication and carbonization, for the fabrication of glass microfluidic channels, using a glass imprinting process. In our previous research, we demonstrated large area glass imprinting at micro and nano scale resolutions using VC molds [28,29]. Since texturing of the VC mold is conducted via thermal replication, a large area mold compatible with glass imprinting can be fabricated at a low cost. Finally, the initial autofluorescnece of the fabricated micro channel was evaluated. To verify the feasibility of the proposed solution, a flow-focusing droplet generator was fabricated using a soda-lime glass microchannel plate and PDMS top plate. 

## 2. Materials and Methods

### 2.1. Fabrication of A Vitreous Carbon Stamp

The VC mold was fabricated via the carbonization of a Furan-based replica, which was itself fabricated via a series of replication steps using PDMS and a furan-based thermal curable polymer. Since the demolding properties of the furan-based resin are extremely poor, it is not feasible to replicate a silicon wafer master pattern directly. The first replication step generated an elastomeric intermediate mold made from PDMS. PDMS has superior mechanical and surface properties, and can transfer a pattern to a Furan-based resin with high fidelity. The intermediate PDMS mold was fabricated via a conventional soft-lithography process using 10 parts of Sylgard 184 (Dow Corning Korea Ltd., Seoul, Korea) and 1 part of a curing agent. The master pattern of the microfluidic structure was fabricated on a 4 inch silicon wafer using a photolithography process that employed SU-8 3050 (MicroChem Co., Westborough, MA, USA) as a photoresist. The height of the master pattern was 40 µm. 

The second replication step was performed using a mixture that included a furan resin (Kangnam Chemical Co. Ltd., Gwangju, Gyeonggi, Korea), p-TSA (p-Toluenesulfonic acid), and ethanol. The furan mixture was poured onto the PDMS mold. Before solidification, the mixture was degassed to remove air bubbles created during mixing. In the first curing process, the mixture was allowed to polymerize naturally over 5 d under atmospheric conditions. Next, thermal curing was performed in a conduction oven at up to 100 °C for 2 d.

**Figure 1:**
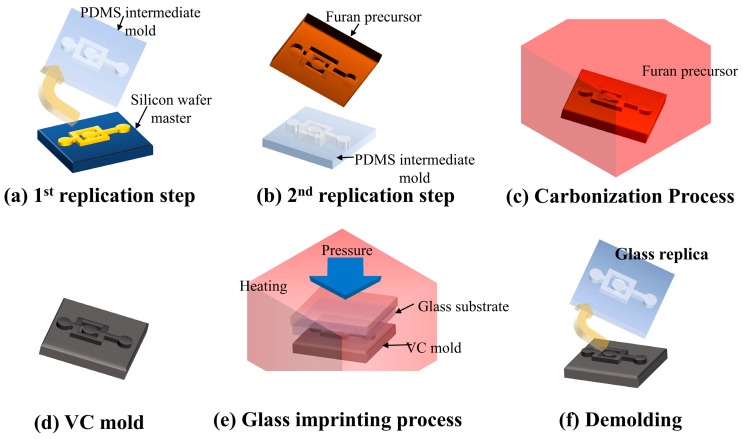
Proposed fabrication method for the glass microfluidic device. PDMS is poly-dimethylsiloxane; VC is vitreous carbon.

The furan precursor synthesized using the aforementioned method was carbonized in a furnace at 1000 °C under N_2_ for 5 d. During this process, pyrolysis phenomena caused shrinkage of the furan precursor to occur. The pyrolysis is when all the molecules in the furan precursor, except carbon, are thermally decomposed and only carbon remains. Molecules which are thermally decomposed in this way include hydrogen and oxygen. The pyrolysis process results in a mass reduction and a volumetric shrinkage. Figure 1a–d shows a schematic diagram of the proposed VC stamp fabrication method.

### 2.2. The Glass Imprinting Process

**Figure 2:**
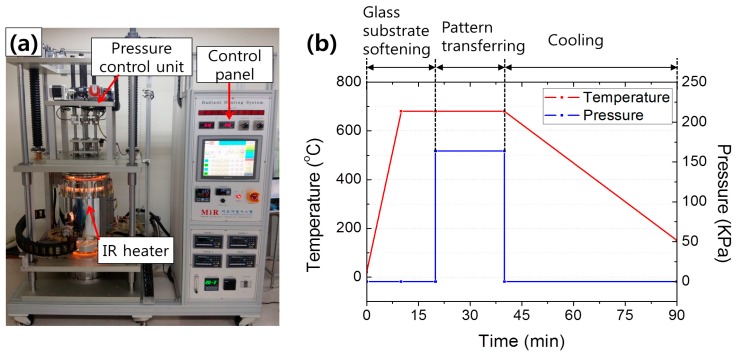
(**a**) Photograph of the glass imprinting system and (**b**) the temperature and pressure conditions during the glass imprinting process.

To evaluate the process of imprinting glass using a VC stamp, a high temperature thermal press system consisting of an infrared (IR) heater, a motor-driven pressure module, and a controller was developed. The system allows precise temperature and pressure control up to 1050 °C and 900 N, respectively. Figure 2a shows the imprinting system. 

The glass imprinting process was performed using this system. A soda-lime glass substrate with a thickness of 3 mm was used as the imprinting material. Figure 1e–f show the schematic flow diagram of the glass imprinting process. First, the VC mold was installed in the middle of the imprinting system and the glass substrate was placed so as to cover the VC mold. To prevent the oxidization of the VC mold during the process, the chamber was maintained in an inert environment via nitrogen purging. The mold and substrate were heated up to the imprinting temperature with a heating rate of 70 °C/min and the temperature was maintained for 10 min in order to obtain uniform temperature distribution. After the holding time, a compression pressure was applied for 20 min. After cooling to room temperature, the glass replica was removed from the VC mold.

**Figure 3:**
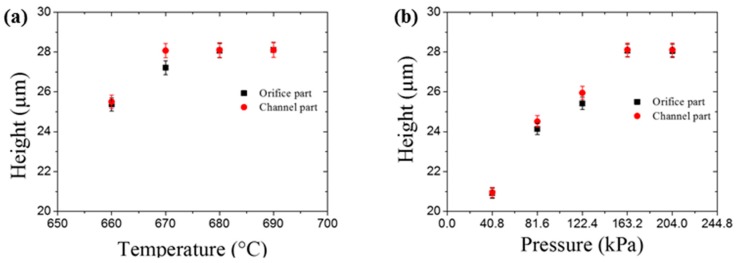
The height of the orifice and channel parts of imprinted glass microfluidic chips in with relation to (**a**) temperature at the same pressure (163.2 kPa) and (**b**) pressure at the same temperature (680 °C).

To obtain the optimum imprinting condition, the effects of imprinting (a) temperature and (b) pressure on the height of microchannel at the orifice and channel regions were analyzed as shown in Figure 3. It clearly shows that the heights of the imprinted glass microchannels increased as the temperature and pressure increased, and that the measured heights reached appropriate values when the temperature was higher than 680 °C and the pressure was higher than 163.2 kPa. Therefore, we selected an imprinting temperature of 680 °C and a maximum imprinting pressure of 163.2 kPa as the optimal conditions.

### 2.3. Sealing The Glass-Based, Droplet-Generating Microfluidic Chip

To examine the feasibility of using the glass replica for microfluidic applications, the glass-based microfluidic pattern was sealed with a flat PDMS block via an O_2_ plasma bonding process. Although sealing was performed with a PDMS block instead of the glass substrate, the purpose of this test was to verify the fidelity of the glass imprinting process. Conventional glass-to-glass bonding solutions such as thermal fusion bonding [30] and anodic bonding [31] are available. Fabrication of all-glass microfluidics may be achieved by combining the glass imprinting process with the aforementioned glass-to-glass bonding methods. We made holes for inlets and outlets on the PDMS block using a biopsy punching tool with a diameter of 1.5 mm. Subsequently, a glass replica and the PDMS block were bonded via O_2_ plasma surface treatment at 18 W for 30 s.

## 3. Results

**Figure 4:**
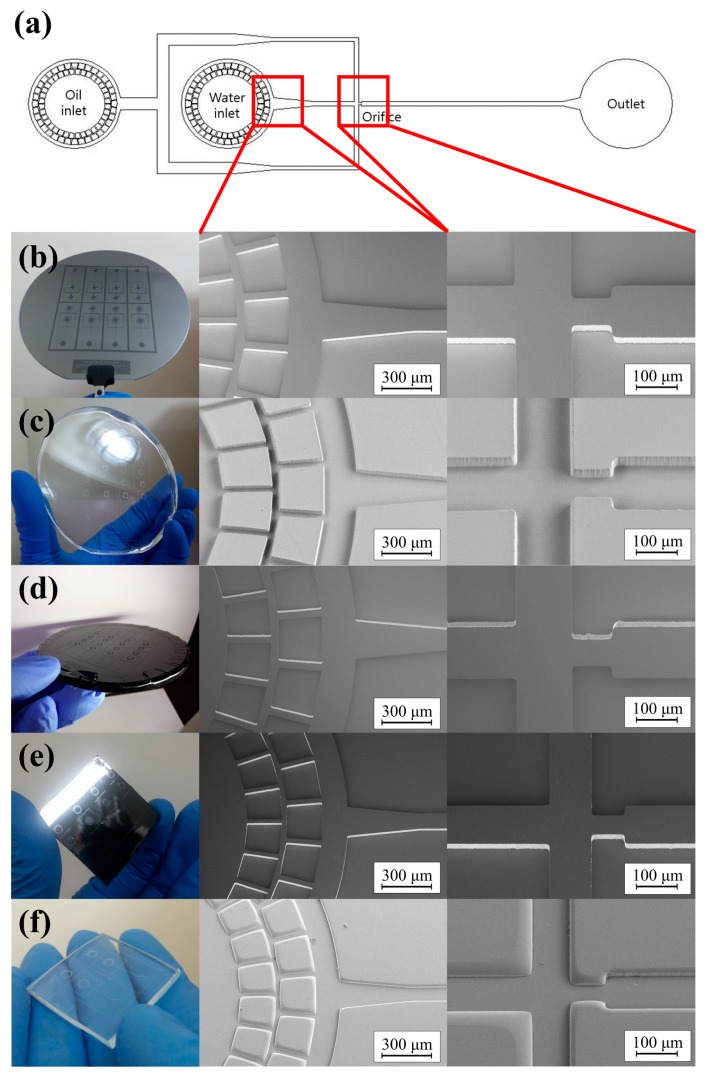
(**a**) Design of the droplet-generating microfluidic chip, and photograph and SEM images of the replicated glass microfluidic structure: (**b**) the silicon master, (**c**) the PDMS mold, (**d**) the furan precursor, (**e**) the vitreous carbon stamp, and (**f**) the replicated glass microfluidic structure.

**Figure 5:**
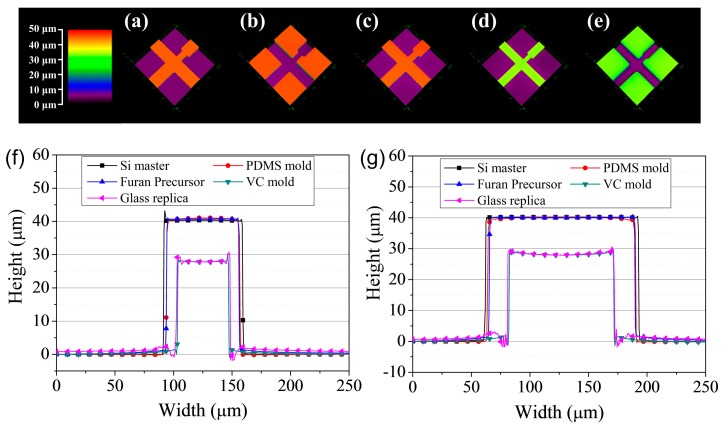
3D microscope measurement results of (**a**) the silicon master, (**b**) the PDMS mold, (**c**) the furan precursor, (**d**) the vitreous carbon stamp, and (**e**) the replicated glass microfluidic structure. Comparison of the surface profiles measured in (**f**) the channel and (**g**) the orifice.

To verify the fidelity of the glass imprinting process, the samples were measured at each step. That is, (a) the silicon master, (b) the intermediate PDMS mold, (c) the furan replica, (d) the VC stamp, and (e) the replicated glass microfluidic structure were each measured. Figure 4 shows photographs and scanning electron microscopy (SEM) images of samples from each step. For quantitative evaluation, measurements were taken using a confocal microscope (OLS-4000, Olympus Co., Tokyo, Japan). Figure 5a–e show the 3D surface profiles of (a) the silicon master, (b) the PDMS mold, (c) the furan precursor, (d) the VC stamp and (e) the replicated glass microfluidic structure obtained by the confocal microscope measurement results. Figure 5f–g show the comparison of the surface profiles measured at (f) the channel and (g) the orifice. As shown in Figure 5f–g, the difference between the surface morphologies of the silicon master and Furan replica is negligible. However, considerable shrinkage occurred during the carbonization process. To analyze the shrinkage characteristic and repeatability of the proposed glass imprinting process with a VC mold, the geometrical properties of three PMDS molds, Furan precursors, and VC molds fabricated from a single silicon master, as well as 9 glass replicas from the three fabricated VC molds, were analyzed as summarized in Table 1. The total shrinkage ratios of the VC were 30.43% for orifice width, 30.17% for channel width, and 30.33% for height. It is clear that the almost isotropic shrinkage occurred in the VC mold fabrication process. The percent coefficient of variance of measured values in the VC mold were less than 1%. This means that the repeatability error of the proposed VC mold fabrication is under 1%, which is acceptable accuracy in microchannel applications. The dimensional difference between the VC molds and glass replicas was negligible because of the similar thermal expansion ratio of VC and glass materials. In addition, the surface roughness of the VC mold must be assessed to determine that it would be acceptable in microfluidic applications. Table 2 shows the channel wall surface roughness as measured in each step. Five different positions were measured on each sample, and the results were averaged. The results show that the deterioration of the surface roughness during VC mold fabrication and the glass imprinting process was negligible. 

Droplets were generated using the chip in order to investigate the applicability of the fabrication method. We prepared an apparatus for the observation of droplet generation. The apparatus included an inverted microscope (CKX-41, Olympus Co., Tokyo, Japan), two precision syringe pumps (Legato 200 & KDS 100, KD Scientific.), and a high-speed camera (CR600x2, Optronis GmbH, Kehl, Germany). Droplet generation was conducted using deionized water as a dispersed phase and fluorocarbon oil (HFE-7500, 3M Co., St. Paul, MN, USA) as a continuous phase. The microchannel surfaces were made hydrophobic via treatment with a commercial reagent (Repel-Silane ES, GE Healthcare Co., Waukesha, WI, USA). Table 3 summarizes the test results with an aqueous phase fixed flow rate of 30 µl/min and 4 different oil phase conditions.

**Figure 6:**
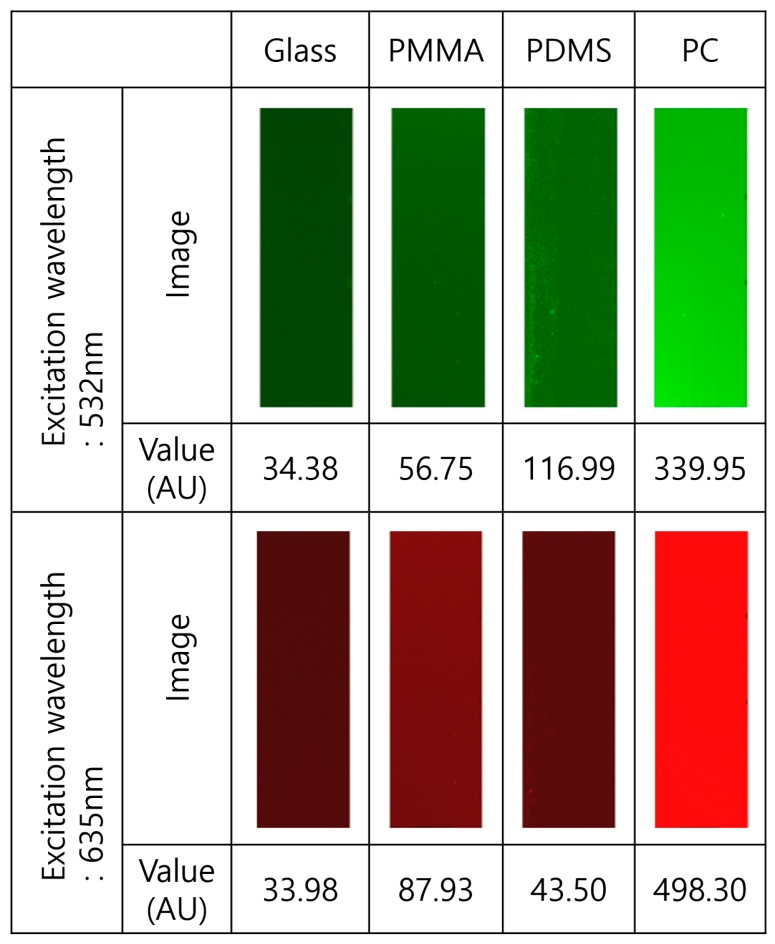
Measurement results for the autofluorescence in different materials commonly used for microfluidic devices; The unit of autofluorescece is absolute unit (AU).

Finally, the initial autofluorescence of the fabricated micro channel structure was measured for two different excitation wavelengths: 532 nm and 635 nm. Those were compared with results from other commonly used materials for microfluidics, including PDMS, Poly(methyl methacrylate) (PMMA) and polycarbonate (PC). Laser-induced fluorescence detection was applied to measure the initial autofluorescence of the microfluidic chips. As shown in Figure 6, the fabricated glass microfluidic device has low initial autofluorescence level. We believe that this glass microfluidics fabrication method could be a powerful solution for the devices used in applications requiring sensitive fluorescence detection at low cost.

## 4. Conclusions

In this study, we developed a glass imprinting process for the manufacturing of glass microfluidic chips. This method uses a VC mold to provide cost-effective microstructure texturing on a glass substrate. The VC mold was fabricated by combining a furan-based thermal replication process with carbonization. The overall geometrical deviation between the silicon master and the VC mold, due to the shrinkage of the furan resin during carbonization, was approximately 30%. Degradation of the surface integrity during carbonization was negligible. Since the shrinkage was isotropic and repeatable (30.31% with less than ~1% error), it can be predicted and could be compensated for by enlarging the initial master pattern. To verify the applicability of this method, a microfluidic chip designed for droplet generation via a flow-focusing structure was fabricated. Droplet generation was demonstrated at various flow rates. We demonstrated that our approach can provide cost-effective glass microfluidic chip fabrication. The fabrication of the all-glass chip via thermal fusion bonding and the practical application is the subject of ongoing research. In addition, the proposed VC mold fabrication and glass imprinting technique will be extended to nanoscale structured devices.

## Figures and Tables

**Table 1:** Comparison of the pattern widths and heights measured after each fabrication process.

		Si Master	PDMS Mold	Furan Precursor	VC Mold	Glass Replica
Orifice width	Mean (µm)	62.53	61.83	61.33	43.50	43.54
	Standard deviation (µm) (coefficient of variance)	- (-)	0.1559 (0.252%)	0.1179 (0.192%)	0.2700 (0.621%)	0.2569 (0.590%)
	Total Shrinkage ratio (from master)	-	0.98%	1.91%	30.43%	30.30%
Channel width	Mean (µm)	127.35	126.22	125.06	88.93	88.87
	Standard deviation (µm) (coefficient of variance)	- (-)	0.0236 (0.019%)	0.2585 (0.207%)	0.1671 (0.188%)	0.2718 (0.306%)
	Shrinkage ratio (from master)		0.89%	1.80%	30.17%	30.22%
Height (µm)	Mean (µm)	40.29	40.13	39.84	28.07	27.91
	Standard deviation (µm) (coefficient of variance)	-	0.0125 (0.031%)	0.0535 (0.134%)	0.1337 (0.476%)	0.1808 (0.648%)
	Shrinkage ratio (from master)	-	0.41%	1.12%	30.33%	30.74%

**Table 2:** Comparison of the roughness generated by each of the fabrication processes.

	Arithmetic Average of the Roughness (Ra, nm)	Root Mean Squared Roughness (Rq, nm)
Si master	1.67 ± 0.52	2.17 ± 0.41
PDMS mold	3.83 ± 1.47	5.17 ± 1.47
Furan Precursor	1.67 ± 0.52	2.17 ± 0.41
VC mold	2.83 ± 0.41	3.67 ± 0.52
Glass replica	5.50 ± 1.76	7.50 ± 3.02

**Table 3:** Comparison of droplet generation frequencies achieved under various flow rate conditions.

Flow Rate of Aqueous Phase (μL/min)	Flow Rate of Oil Phase (μL/min)	Generation Frequency (Hz)	Captured Image from the Movie Captured by High Speed Camera
30	2.5	469	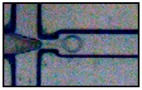
	5	872	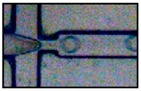
	10	1588	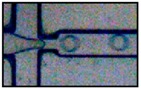
	15	2521